# Living 20 years with perineal colostomy and dynamic graciloplasty – a case report discussing the role of this approach

**DOI:** 10.1007/s00384-025-05036-z

**Published:** 2026-01-03

**Authors:** Harald Rosen, Christian G. Sebesta, Marie Sebesta, Christian Sebesta

**Affiliations:** 1 Center of Surgery, Sigmund Freud University Vienna, Freudplatz 3, 1020 Vienna, Austria; 2 Science Center Donaustadt, Langobardenstr. 122, 1220 Vienna, Austria; 3 Clinic Donaustadt, Department of Oncology, Langobardenstr. 122, 1220 Vienna, Austria

**Keywords:** Total anorectal reconstruction, Graciloplasty, Perineal colostomy, Rectal cancer, Case report

## Abstract

**Background:**

Despite advances in neoadjuvant therapies and surgical techniques, abdominoperineal excision of the rectum (APER) is still necessary in a considerable number of cases, often requiring the creation of a permanent colostomy, which can significantly impact a patient’s quality of life (QOL). Total anorectal reconstruction (TAR) with dynamic graciloplasty has emerged as a reconstructive option for patients undergoing APER, aiming to restore continence by avoiding a permanent abdominal colostomy and improving quality of life. However, this approach presents several challenges, including technical complexity and variable long-term outcomes.

**Case report:**

We present the case of a 34-year-old female patient who underwent APER with extended resection (rectum and vaginal wall) due to low rectal adenocarcinoma infiltrating the posterior vaginal wall. Following a prolonged postoperative course and the decision against living with an abdominal colostomy, the patient underwent secondary TAR with reconstruction of the posterior vaginal wall and dynamic graciloplasty in 2001. The procedure included creating a neorectum using a myocutaneous flap for vaginal reconstruction and a gracilis muscle wrap with neurostimulation as a neosphincter. Despite early postoperative complications, the patient achieved satisfactory continence with regular transanal irrigation and lived with the reconstruction for over 20 years. In 2024, the patient returned for management due to the obsolescence of her neurostimulator, which was subsequently removed without deterioration in her continence function.

**Conclusion:**

This case highlights the complex and prolonged management challenges associated with TAR and dynamic graciloplasty for patients with severe anorectal dysfunction following APER. While dynamic graciloplasty has been shown to offer some level of continence in patients with faecal incontinence, the need for additional interventions, such as regular irrigation, is often required to maintain quality of life after TAR following APER. The durability of this reconstructive approach and the patient’s long-term satisfaction underline its potential as a viable, though technically demanding, alternative to conventional colostomy in selected patients. However, the role of electrically induced muscle fiber transformation (“dynamic graciloplasty”) needs to be discussed.

## Introduction

Abdominoperineal excision of the rectum (APER) remains a critical treatment option for patients with low rectal cancer, particularly when sphincter-preserving surgery is not feasible due to tumor location, size, or involvement of the sphincter complex [1, 2]. Despite advances in neoadjuvant therapies and surgical techniques aimed at enhancing the possibility of sphincter preservation, APER continues to be indicated in a significant proportion of cases [1, 2]. The incidence of APER varies worldwide, with some studies suggesting that up to 20% of rectal cancer patients may require this procedure, necessitating the creation of a permanent colostomy [1]. A permanent colostomy can profoundly impact a patient’s quality of life, affecting not only the physical and practical aspects of stoma care but also the psychological and social dimensions. As a result, the decision to perform APER and create a permanent colostomy is carefully weighed against the potential benefits of alternative reconstructive approaches.

Total anorectal reconstruction (TAR) is a surgical procedure that aims to restore continence and improve quality of life in patients who have undergone APER or experienced traumatic loss of anorectal function [3–6]. The combination of perineal colostomy and graciloplasty has emerged as a viable option for reconstructive surgery in these patients. This approach, however, comes with its own set of challenges and complications that can significantly impact long-term outcomes [3–6].

The initial reports on dynamic graciloplasty were published by Williams et al. in 1991, who described the use of the gracilis muscle, transposed and electrically stimulated, to act as a neosphincter [3]. Following this, the procedure gained traction with subsequent refinements and studies demonstrating its feasibility and effectiveness in restoring continence [3–6]. Further studies have tried to evaluate the potential impact of TAR in patients following APER either in a primary fashion (i.e. as a synchronous procedure) but also as a secondary operation years after APER when a recurrence-free situation and/or a rejection of a life with a colostomy from the patient’s side was accepted, although this specific procedure is technically more demanding and associated with a higher rate of morbidity.

Despite advances in surgical techniques and postoperative care, the long-term management of patients living with a perineal colostomy and graciloplasty remains complex. Geerdes outlined the challenges faced by patients undergoing anorectal reconstruction, including the risk of infection, muscle fatigue, and the psychosocial impact of living with modified anatomy [5].

This case report presents a patient who has lived with a perineal colostomy and graciloplasty for more than 20 years, providing a unique perspective on the position of TAR in the spectrum of colorectal surgical interventions. By examining the long-term clinical and functional outcomes, this report aims to shed light on the viability of TAR with dynamic graciloplasty as a durable solution for patients with anorectal dysfunction and to discuss its place in current surgical practice.

## Case report

This case report was prepared in accordance with the CARE (CAse REport) reporting guidelines.

In February 1999, a 34-year-old female patient presented to our unit for the treatment of histologically proven adenocarcinoma of the low rectum infiltrating the posterior wall of the vagina. Due to the extent of the local growth, the decision was made to perform a terminal left-sided abdominal colostomy with closure of the remaining rectum before she could be referred for neoadjuvant radiotherapy.

In May 1999, clinical and radiologic evaluation revealed persisting involvement of the posterior vaginal wall and the levator ani muscle, leading to the decision to perform an extended APER with hysterectomy and resection of the posterior vaginal wall. Histological evaluation revealed a stage yT4 N0 (0/25) G3, with the uterus showing no tumor involvement. Multiple biopsies from the lateral pelvic floor (i.e. remnants of the levator muscle following APER) showed no evidence of malignancy.

The postoperative course was prolonged by delayed healing of the perineal and vaginal wounds, but the patient was eventually discharged to her hometown.

Since the patient remained free of recurrence until 2001 and did not tolerate life with an abdominal colostomy and a partially resected vagina, it was decided to proceed to a secondary total anorectal reconstruction and reconstruction of the posterior vaginal wall.

In October 2001, laparotomy was performed, and following adhesiolysis, the abdominal stoma was resected. The perineal scar was excised, and a tunnel through the pelvic and perineal scars was created for a pull-through of the descending colon to serve as a neorectum.

Following this, a myocutaneous flap including the right gracilis (“island flap”) muscle was prepared and transposed to the vagina, serving as a reconstruction for the previously resected posterior vaginal wall.

Then the left gracilis muscle was mobilized and wrapped around the descending colon in a typical manner (“split sling technique”) as previously published [6].

Two stimulation electrodes were introduced near the most proximal neurovascular bundle of the left gracilis muscle and connected to a neurostimulator (INTERSTIM I, Medtronic, Dublin, Ireland) implanted in the subcutaneous tissue of the left lower abdomen.

The procedure was completed by the construction of a protective ileostomy.

The postoperative course was complicated by an injury to the right ureter, which required revision by a psoas hitch procedure on the third postoperative day.

Following this, the patient did not experience any further problems, and the electrical stimulation program for muscle fiber transformation was initiated as described elsewhere [6], followed by the reversal of the protective ileostomy 6 weeks later.

Once muscle fiber transformation was accomplished and tetanic contraction of the neosphincter was feasible, the patient was instructed to handle the neurostimulator using a magnet or a remote control, respectively, to achieve regular defecation.

However, despite a squeeze pressure (during stimulation) of 100 mm Hg and a resting pressure (without stimulation) of 60 mm Hg, the patient complained of incontinence episodes mainly due to an inability to empty her colon sufficiently.

Therefore, we instructed her to perform regular transanal irrigation (TAI) using a 28 French Foley catheter with a 10 ml balloon and 100 ml syringes, irrigating her rectum with 1000 ml of tap water every 24 h.

Following this, she achieved a satisfying continence situation with no further episodes of fecal incontinence and returned to her hometown, where further follow-up was performed.

In July 2024 (25 years following her first presentation at our unit), she was referred to us again as her neurostimulator had lost its energy and was no longer produced. Furthermore, the new generation of neurostimulators available (INTERSTIM III, Medtronic, Dublin, Ireland) could not be connected to her stimulation electrodes.

**Fig. 1:**
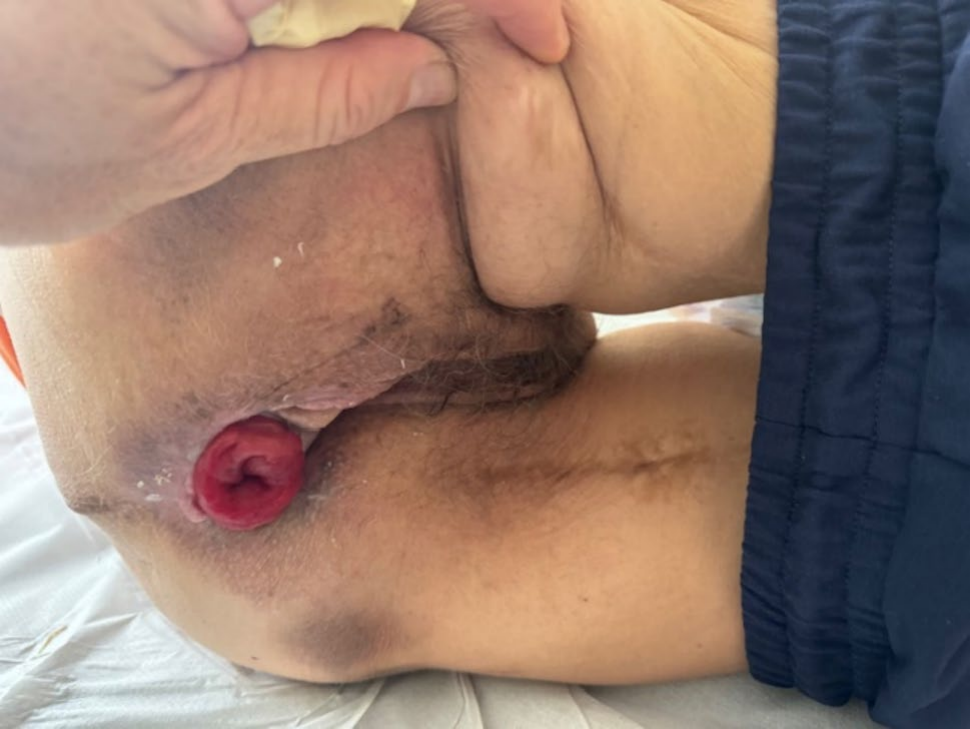
Perineal colostomy and reconstructed vagina following total anorectal reconstruction. Follow-up after more than 20 years

At her visit to our clinic, she showed a perfectly healed perineal stoma and a vaginal reconstruction, which allowed her normal sexual activity (Fig. 1).

According to her history, she was performing transanal irrigation using a total volume of 400 ml of water every 48 h, achieving a situation without any episodes of fecal incontinence. However, she was using a pad regularly to prevent any staining of her underwear from mucus of the perineal stoma.

Since she mentioned having realized the end of life of her neurostimulator already 2 years prior without any deterioration of her continence function, it was decided to explant the neurostimulator from its pocket under local anesthesia, leaving the electrodes in place.

## Discussion

This case report highlights the complex, long-term management of a patient living with a perineal colostomy and graciloplasty for 20 years, offering valuable insights into the challenges and outcomes associated with total anorectal reconstruction (TAR). Dynamic graciloplasty, introduced in the early 1990 s, has been utilized as a reconstructive option for patients with severe anorectal dysfunction, particularly following abdominoperineal resection (APER) [1–6] Although our own experiences with 32 patients published in the nineties of the last millennium showed similar positive results (especially after the introduction of TAI) this patient was the only case in our series who required a synchronous reconstruction of the vaginal wall. Despite its potential to provide a certain level of continence and improve quality of life, the procedure is associated with a range of complications and varied success rates, which are evident in this case and supported by the literature [4, 6].

The formation of a perineal colostomy as an alternative to the “classic” abdominal colostomy has been described repeatedly in the literature using various techniques like the pseudocontinent smooth muscle wrap or a mere formation of a perineal stoma [7–9]. Similar to our patient, all publications dealing with this approach reported the need for irrigation to achieve satisfying pseudocontinence for the patients—either antegrade via an appendicostomy (Malone procedure) or by retrograde irrigation, respectively. In a review of the current aspects of total anorectal reconstruction, Inglin and coworkers [1] as well as by Portier [10], and Farroni [11], who all achieved satisfying QOL as well as pseudocontinence by this approach.

Lahnaoui et al. compared in a prospective study 49 patients who underwent APER and received either an abdominal colostomy (*n*: 16) or a pseudocontinent perineal colostomy (*n*: 33) [8]. While similar rates of early perineal complications were observed between the two groups (*p *= 0.49), the readmission rate was higher in the abdominal stoma group due to perineal sepsis (*p* = 0.09). QOL analysis at 6 months revealed that patients with a perineal colostomy had a higher global health status (*p* = 0.006), better physical functioning, and reported fewer symptoms of flatulence and fecal incontinence (*p *= 0.001) [8].

Based on the available literature, it is widely accepted that there is only a small and distinct subset of patients who will be candidates for the formation of a perineal colostomy following APER or anorectal trauma despite the need for regular transanal irrigation and/or the necessity to wear pads for mucous discharge from the perineal colostomy. It has been demonstrated that geographic location and religious factors have a strong impact on the QOL outcome of patients who undergo the formation of a permanent abdominal colostomy [12].

In our case report, a young woman who had already lived for a significant time with an abdominal colostomy and a partially resected vagina opted for a technically demanding procedure, thus reaching a significant improvement in her QOL for more than two decades.

Is there an indication for dynamic (stimulated) graciloplasty today?

Muscle fiber transformation by electrical stimulation of the gracilis muscle to create a neosphincter capable of tetanic contraction was introduced in the 1990 s [1]. Although sufficiently high sphincter pressures (proven by manometry) could be measured, the functional results following TAR were not satisfying, as all patients reported severe emptying disorders leading to repeated episodes of incontinence. Only after the introduction of irrigation (as already mentioned above) could a pseudo-continence with a significant improvement in QOL be achieved [5, 6].

This observation aligns with findings in the management of low anterior resection syndrome (LARS) following low anterior resection [13]. The introduction of regular TAI has markedly improved this problem, as published recently in various studies [13].

It must be accepted that muscle fiber transformation by electrical stimulation does not add any benefit during TAR which is in accordance with the observation that our patient most probably did not have any electrical stimulation of her muscle for more than 10 years before we saw her again (as the average longevity of the stimulator is around 10 years). Furthermore, it must be noted that the stimulation electrodes used in this patient are no longer available, thus making electrical stimulation of the gracilis muscle not feasible anymore [14].

However, the placement of a muscle wrap (either gracilis or gluteal muscle) could serve as an additional tool to support TAI and prevent leaking during the irrigation process. Furthermore, it could help against a common complication following the formation of a perineal colostomy, i.e., stoma prolapse and/or perineal hernia, which are frequently observed due to the large muscular defects during APER [15].

While some authors have advocated the use of surgical meshes during the formation of a perineal colostomy [15], we believe that a muscle wrap could help to reconstruct some volume of the resected pelvic floor muscles, thus avoiding or at least reducing the occurrence of a perineal hernia with or without colostomy prolapse.

## Conclusion

Total anorectal reconstruction by colonic pullthrough procedure and neosphincter reconstruction by graciloplasty is a possible therapeutic option for selected patients who want to avoid an abdominal colostomy after APR. Although electrical stimulation of the gracilis muscle showed no additional benefit in the long term the formation of a perineal colostomy together with regular transanal irrigation was able to provide an excellent quality of life in this selected patient population.

## Data Availability

Data are available on request with the first author H.R.
